# Pilot Scale Production of Precast Concrete Elements with Wood Biomass Ash

**DOI:** 10.3390/ma14216578

**Published:** 2021-11-02

**Authors:** Jelena Šantek Bajto, Nina Štirmer, Sonja Cerković, Ivana Carević, Karmen Kostanić Jurić

**Affiliations:** 1 Department of Materials, Faculty of Civil Engineering, University of Zagreb, Fra Andrije Kačića Miošića 26, 10000 Zagreb, Croatia; nina.stirmer@grad.unizg.hr (N.Š.); sonja.cerkovic@grad.unizg.hr (S.C.); ivana.carevic@grad.unizg.hr (I.C.); 2 Tomting 2010 Ltd., Novakova 26, 10000 Zagreb, Croatia; karmen.kostanic@tomting.hr

**Keywords:** wood biomass ash, cement-based composites, low-carbon cement replacement, compressive strength, durability

## Abstract

Downsizing fossil fuel dependence and greenhouse gas emissions is at the forefront of a sustainable future. The expansion of renewable energy while striving to minimize dependence on fossil fuels has led to biomass taking the lead among renewable energy sources, with wood having the broadest application. Along with the growing trend of using biomass as a renewable energy source, the combustion of wood biomass results in wood biomass ash (WBA), leading to compelling amounts of waste. In this study, the technical feasibility of fly WBA from different Croatian power plants was analyzed to evaluate its potential use in precast concrete drainage elements and curb units. By implementing a performance-based design, the influence of various factors in thermal processing of wood biomass was investigated, together with a detailed characterization of WBA in order to assess the feasibility of using WBA as a secondary raw material in a large-scale industrial batching plant. The compressive strength and durability properties (water absorption, permeability, and freeze–thaw resistance) of concrete mixtures with WBA as a replacement for 15 wt% cement were evaluated and compared with the precast concrete manufacturer’s technical requirements. The main concerns identified were compositional inconsistency of WBA, workability downturn, delay in initial reactivity rate, and increased water absorption. Concrete with WBA based on a circular design has been found to be a viable solution to cement depletion, stepping up from recycling to reuse of industrial waste.

## 1. Introduction

Modern society is currently facing widespread, rapid, and worsening climate breakdown, while environmental degradation is having a nearly irreversible impact on people, ecosystems, and livelihoods around the world [1]. According to the latest data provided by the Intergovernmental Panel on Climate Change (IPCC) [2], global temperatures are likely to exceed the threshold of 1.5 °C during the 21st century if anthropogenic emissions of carbon dioxide (CO₂) and other greenhouse gasses are not drastically reduced in the near future. Atmospheric warming is dangerously close to spinning out of control, and if the pollution trend continues ramping up, the world should expect even greater climate variability in the upcoming decades, if not centuries. Rapid actions to cut down greenhouse gas emissions might somewhat curb some impacts that are clearly human-induced, but others are already inevitable. The alarming heat waves, giant hurricanes, droughts, and other weather extremes that are already occurring will only get worse [2,3,4]. Although the COVID-19 pandemic has led to the largest drop in global emissions ever, CO_2_ levels in 2020 hit 417 ppm, the highest recorded level in human history [5,6]. This underpins Europe’s plan to attain climate neutrality by 2050, the backbone of the European Green Deal. For this reason, in July 2021, the European Commission presented the “Fit for 55” package, which reaffirms Europe’s ambition to cut down greenhouse gas emissions by at least 55% compared to pre-industrial levels and proclaims the EU’s climate neutrality target enforceable [7]. If the European Green Deal is to become a realistic and tangible experience for all, Europe has to move to a new, innovation-driven model and decarbonize energy-intensive industries, such as the cement industry. Every sector faces major challenges in reducing emissions, and the cement industry is no exception. As a massive emitter of CO_2_ in the world, the cement industry could, therefore, be responsible for up to 8% of global CO_2_ emissions [8,9]. Moreover, the negative impacts of using cement in concrete are associated with abundant depletion of raw material sources, which contradicts the principles of a circular economy [10,11,12,13]. As pointed out in the reports of the International Energy Agency [14], in the interest of creating an energy-saving framework, the cement industry should attempt to use sustainable alternatives for fuels and resources. Efforts by developing countries to ensure homes for all and major infrastructure projects boost cement production every year. At the same time, without efforts to reduce the demand for cement, the annual production is projected to increase moderately by 2030 [7]. Tackling these issues has brought supplementary cementitious materials (SCMs) into the limelight. The use of environmentally friendly SCMs to partially replace cement in concrete is a potential way to support downsizing CO_2_ emissions from cement production. Therefore, lowering the global cement demand can be supported by using locally sourced by-products, such as wood biomass ash (WBA). By superimposing an interdisciplinary approach to identify WBA as a valuable resource for the 21st century construction industry, the problem of industrial waste, while conserving natural resources, would be solved. With increasing awareness and commitment to combat the climate crisis, more and more biomass power plants are coming on stream.

An important step towards creating an energy system fit for a green paradigm shift is being taken by extending the overall binding target from the current 32% (set in the Renewable Energy Directive (REDII)) to a new 40% share of renewables in the EU energy mix [15,16]. In order to reach these ambitious goals, Europe aims to phase out coal combustion, largely depending on the capacity of renewable energy sources to fill in the gap left by the coal downturn [17]. The updated Renewable Energy Directive suggests that bioenergy and biomass could make a valuable contribution in creating a low-carbon energy system with a share of 18% of the total energy supply in 2050 [7], with a presumption of a sustainable handling of biomass [18,19]. The industrial use of bioenergy is expected to increase almost threefold to 24 EJ by 2060, providing almost 14% of industrial energy demand [20]. Furthermore, materials derived from biomass will play a key role in the transition to a circular economy, which must be based on sustainable consumption and production and promote waste recycling [21,22]. 

As wood biomass is identified as a CO_2_-neutral energy source because it releases almost as much CO_2_ during combustion as it absorbs during its growth, it is one of the most important sources of biomass for energy production in the EU, with a majority share of 59% in total renewable energy [18,19,23]. Considering that the combustion of 1 t of wood biomass results in about 3% WBA [24], the expansion of wood biomass power plants already results in sizable amounts of wood biomass ash (WBA). While these figures are clearly signaling the urgency of strategic foresight in waste management, the continuing routine in Europe is based on the disposal of WBA in landfills, usually without any form of control, resulting in additional costs and risks to the environment. Small amounts of WBA are used as soil amendment/fertilizer, but this is only legal in countries that have regulations for recycling WBA for this purpose. Consequently, the existence of illegal landfills is also a growing global problem [25,26,27,28,29,30,31]. With the adoption of the new “Circular Economy Package”, as well as the enhanced Directive 2018/851 on waste and Directive 2018/850 on the landfill of waste, the EU is resolutely committed to minimizing the landfilling of waste, as well as its recovery [32,33]. As reported by [34], about 10 million tons of biomass fly ash are generated in the electricity industry alone. Given the already existing deficit of landfill sites and the strict EU landfill directives, the cost of landfilling will undoubtedly increase in the future. Moreover, WBA consists of very fine particles that can be easily transported through the air and, consequently, cause health problems related to the respiratory system of the population living near the landfill [35]. In addition, when waste ash is landfilled in non-designated sites, there is also the problem of groundwater pollution due to leaching of heavy metals from WBA or rainwater infiltration, all of which have a negative impact on the ecological system [36]. Therefore, by binding the heavy metals from WBA in the cement matrix, the use of WBA in low-carbon concrete contributes to the production of an environmentally friendly and cost-effective alternative cementitious material [37]. To verify an optimum share of WBA as a cement substitute, which has exhibited promising pozzolanic properties, a compromise between the amount of WBA and the downgrade of concrete properties is needed [38,39,40,41]. Ergo, this “novel” concrete containing waste material simultaneously solves the problem of industrial waste, reduces the amount of cement produced, and, consequently, reduces energy consumption and product costs [42,43,44].

Even supposing that WBAs can be used effectively in cement composites, as previous research studies have reported [25,38,39,45,46], there remains a need for comprehensive, long-term, micro-level verification of WBAs to assess the degree of resulting property degradation. While current eco-innovations in manufacturing tend to focus primarily on technological advances, changes on an institutional level often need to complement the necessary technological ones to foster their development.

On the other hand, according to the Croatian National Environmental pollution register, WBA is classified as waste, and its free recovery in construction is currently not regulated as it is for coal fly ash. In order to be competitive with unsustainable, conventional building materials, it is essential to outline the guidelines and standards required for a wider application of WBA in the construction sector. Therefore, showcasing and promoting the health benefits of reusing WBA instead of merely landfilling has been identified as a strategy to promote a positive approach to the acceptance of unconventional materials.

**Figure 1:**
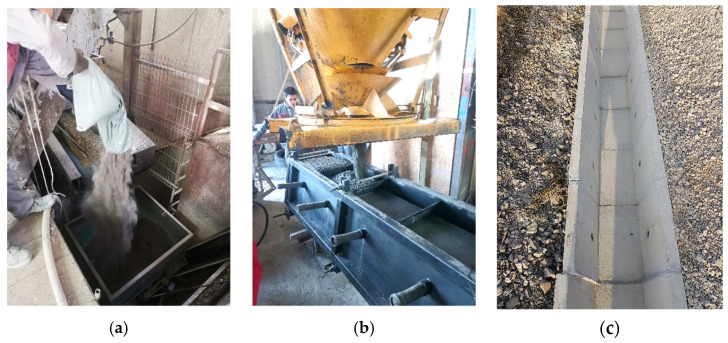
Fabrication of a pilot-scale prototype in an industrial setting: (**a**) manual addition of WBA during concrete mixing; (**b**) filling of curb molds; (**c**) prefabricated drainage elements.

The aim of this study was to find the possibilities for reusing waste WBA in the production of concrete products. For this purpose, the production and testing of concrete with different WBAs in fresh and hardened states were carried out in close cooperation with a precast concrete manufacturer. The results of the tests of mixtures developed under laboratory conditions will be used for prototype production to evaluate the feasibility of using WBAs in an already established plant and to implement a full-scale commercial process, Figure 1. As a result, existing concrete production facilities could spin off new sustainable products at a lower cost. To promote the production of concrete products with alternative materials, recommendations are made for further investigations in line with the research findings aimed at commercializing the innovative products in the global market.

## 2. Materials and Methods

The experimental part of the research aimed to determine a suitable type of WBA that can be used as a partial cement replacement in concrete production, considering the physical properties and chemical composition of different fly WBAs, in order to gain a comprehensive insight into the effects of WBA on the mechanical and durability properties of cementitious composites. A total of 6 concrete mixes were prepared—5 concrete mixtures by replacing cement with 15 wt% WBA and 1 reference control mix, which did not contain any WBA. All concrete mixes with WBA as a replacement for the cement component were produced in the precast concrete manufacturer’s production plant. Based on the previously identified workability issues [25,47], a 15 wt% cement replacement was estimated to be the maximum achievable when using WBA. Moreover, the authors of previous studies confirmed that a cement replacement ratio of 10–15 wt% by WBA is the maximum applicable proportion owing to the abundant amount of free CaO and free MgO detected in WBA samples [25,48,49].

The concrete specimens were prepared by mixing Portland cement CEM I 42.5 R, i.e., binder mixtures of cement and WBA, crushed stone aggregate, and potable water, complying with EN 206 [50]. Equal proportions of superplasticizer and air-entraining agent, dosed in relation to the weight of binder, were maintained in all concrete mixtures. Five WBAs used in this study were collected from power plants in Croatia that use untreated wood chips as fuel, while applying the following combustion technologies: biograte, grate combustion, and grate and pulverized fuel combustion. The types of biomass fuel used in the power plants are pure wood chips, residues from wood harvesting, and waste from the wood industry, with the most common wood species being beech, oak, and hornbeam.

### 2.1. Wood Biomass Ash

The wood biomass fly ash used in the experiment was used as collected, without any prior treatment. A visual evaluation of the samples was performed to sort out ashes that would not be acceptable for the specific application due to impurities in the samples. However, it should be kept in mind that this kind of preliminary assessment of the WBA samples may have varied due to sampling, which is one of the major drawbacks of these materials. The visual assessment of the samples and the general characteristics of the power systems, i.e., combustion type and temperature and type of biomass used, are presented in Table 1. All of the power plants from which the WBA samples were collected use only wood biomass as fuel, without co-firing with fossil fuels. Although the primary influence on the chemical and physical properties of WBA is derived from the type of biomass used in the power plants, combustion technologies also influence the chemical composition of WBA [17,26]. In the power plants themselves, WBA is stored in closed containers where the ash does not come into contact with the atmosphere, but which are not sealed, so the carbonization process and the influence of moisture are possible. Therefore, WBAs ought to be collected and stored immediately upon collection in closed containers to avoid pre-hydration and carbonization. Adequate storage of WBA would be a starting point for boosting the use of WBA in cementitious composites and highlighting the beneficial cementitious properties of WBA [51]. These three main groups (raw material, combustion technology, and storage) of influences straightforwardly meddle with the applicability of WBA as a cement replacement [25,52,53]. 

### 2.2. Concrete Mix Design

The concrete mix design was outlined to demonstrate production, i.e., pilot production of precast concrete using WBA, as per the concrete manufacturer’s requirements for precast concrete elements. Concrete mixes with 15 wt% WBA (designated as CM1, CM2, CM3, CM4, CM5) were prepared by replacing the cement type CEM I 42.5 R. A control mix (designated as M0) was prepared without WBA. The proposed designation for the concrete mixes was CM*i*, where *i* is associated with the number of WBA sample. All concrete mixes were prepared with a maximum aggregate grain size D_max_ = 16 mm. The water/binder ratio used (w/b = 0.44) was constant in all concrete mixes. The chemical admixture, a superplasticizer, was added to each mix at 0.5 mass percent of the binder, while the air entraining admixture was added at 0.1 mass percent of the binder (Table 2).

### 2.3. Methods

The physical and chemical properties of each WBA type were determined prior to the preparation of the concrete mixes, Table 3. The calorimetric analysis of the WBA samples, i.e., the measurement of the heat of hydration of the mixed pastes, was carried out on an 8-channel TAM Air isothermal calorimeter (model 605,000) according to EN 196-11:2019 [54]. The heat of hydration was measured for 7 days at 20 °C, where the ratio of water to cementitious materials was 0.5 and 15 wt% of ordinary Portland cement was replaced with WBA. About 20 g of the paste samples were mixed for 2 min and 10 g of the sample was then placed in a sealed ampoule and lowered into the calorimeter conditioned to 20 ± 0.05 °C. Laser diffraction analysis of particle size was performed using a Shimadzu SALD-3101 analyzer. Samples were dispersed in an air stream at a pressure of 0.4 MPa. The WBAs were sieved through 1 mm sieves to remove impurities and coarser fractions present in certain samples. Scanning electron microscopic (SEM) analysis was performed using JEOL’s JSM-IT200 microscope.

Test methods for assessing concrete properties in fresh and hardened states are listed in Table 4.

## 3. Results and Discussion

### 3.1. WBA Assessment

The chemical composition of the WBAs and cement used in the concrete mixes were compared with the criteria for coal fly ash according to the standard EN 450-1:2013 [55], Table 5. According to the results of chemical analysis, the properties of WBA differ significantly from those of coal fly ash, a traditionally known and widely used mineral admixture [37]. It is also important to emphasize that the chemical composition of WBA can vary significantly depending on the raw material and combustion technology [26,56,57,58]. The standard EN 450-1 addresses only the use of fly ash obtained by co-combustion of wood biomass with fossil fuels. Therefore, the existing regulations for the use of fly ash in cement (EN 450-1) can be applied to WBA only as guidelines for the content of substances that negatively affect the mechanical properties and durability of concrete. Loss on ignition (LOI) averaged 12.78% for all WBAs, which is higher than the maximum value of 9.0% specified in the standard EN 450-1. WBA2 (19.7%) and WBA4 (24.1%) exceeded the LOI limit of 9.0%. However, WBA1 (6.2%), WBA3 (8.8%) and WBA5 (5.1%) met the specified criterion. It is considered that the high value of LOI indicates that WBA contains a significant amount of unburned carbon that reduces the pozzolanic activity itself. In this case, the unburned particles present in WBA diminished its pozzolanic reactivity, which was reflected in the high LOI content [59]. Therefore, a large part of the organic content, expressed as LOI, did not contribute to the compressive strength, i.e., to the mechanical properties [46]. The CaO dominated in all WBA samples, with an average of 45.33%, except in the WBA5 sample where SiO_2_ prevailed with 44.90%. Some authors have reported that WBAs with high calcium content exhibit hydraulic properties, while the contribution of pozzolanic reaction is secondary and slow [47]. The WBA1 (5.56%) and WBA2 (4.57%) samples did not meet the criteria for MgO content less than 4.0 wt%. Large amounts of CaO and free MgO can negatively promote the occurrence of volume deformation (swelling) during the hydration process and consequently lead to cracking [60,61]. Based on the general characteristics of the plants listed in Table 1, no conclusion can be drawn as to which input parameter of the power plant caused the higher SiO_2_ content in the WBA5 sample. However, it is claimed that various parameters, such as biomass type, combustion technology, biomass combustion temperature, collection site, and storage, strongly influence the chemical compounds of WBA [25,50,57,58,59]. Chemical analysis revealed the average content of the most abundant oxides in the WBA (Table 5), with CaO (45.33%) dominating, followed by SiO_2_ (26.52%). The literature review also revealed a predominance of CaO, followed by SiO_2_ and K_2_O [26,62,63]. The sulfate content (SO_3_) ranged from 1.06% (WBA4) to 6.93% (WBA1). Only WBA3 and WBA4 with 1.84% and 1.06% met the maximum sulfate content criterion of 3.0%. The alkali content, Na_2_O_eq_ = Na_2_O + 0.658 K_2_O, given according to EN 196-2:2013 [64], was found to be less than 5.0% in samples WBA3 (3.71%) and WBA4 (3.22%). The total P_2_O_5_ content was less than 5.0% for all WBAs. The sum of pozzolanic oxides in the tested WBAs ranged from 21.19% (WBA2) to 58.81% (WBA5), which is less than the required 70%, indicating that the WBAs are hydraulically active or inert materials. A hydraulic balance greater than 1.4%, determined by the formula (CaO + MgO)/SiO_2_, also suggests possible hydraulic reactivity of WBA [49,65]. According to some authors, higher alkali content in WBAs may affect the more porous structure of cement composites and, consequently, reduce the mechanical and durability properties. Moreover, the release of alkali into the pores of the cement matrix may lead to the development of alkaline silicate reaction (ASR), i.e., volume deformations of the concrete [66].

**Figure 2:**
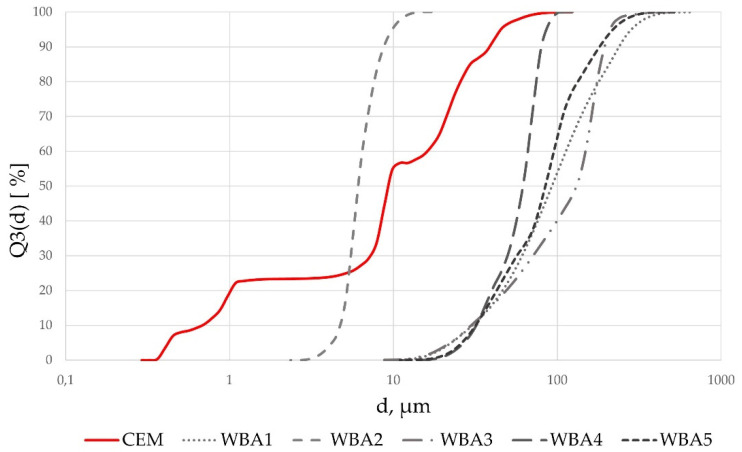
Particle size distribution of WBA and cement samples.

The particle size distribution of WBA samples and cement is shown in Figure 2. The particle size of fly WBAs was above 10 µm for all WBAs, except for WBA2. From the obtained results, it can be seen that WBA2 had the finest distribution, i.e., the percentage of particles smaller than 10 µm is 94.98%. The physical properties of WBA and the replaced cement in terms of density and median d_50_ for particle distribution are shown numerically in Table 6. It was observed that, on average, 50 vol% of WBA ranged from 6.10 µm (WBA2) to 129.52 µm (WBA3). The density of WBA ranged from 2.21 g/cm^3^ (WBA5) to 2.67 g/cm^3^ (WBA1), all lower than the density of the cement used.

**Figure 3:**
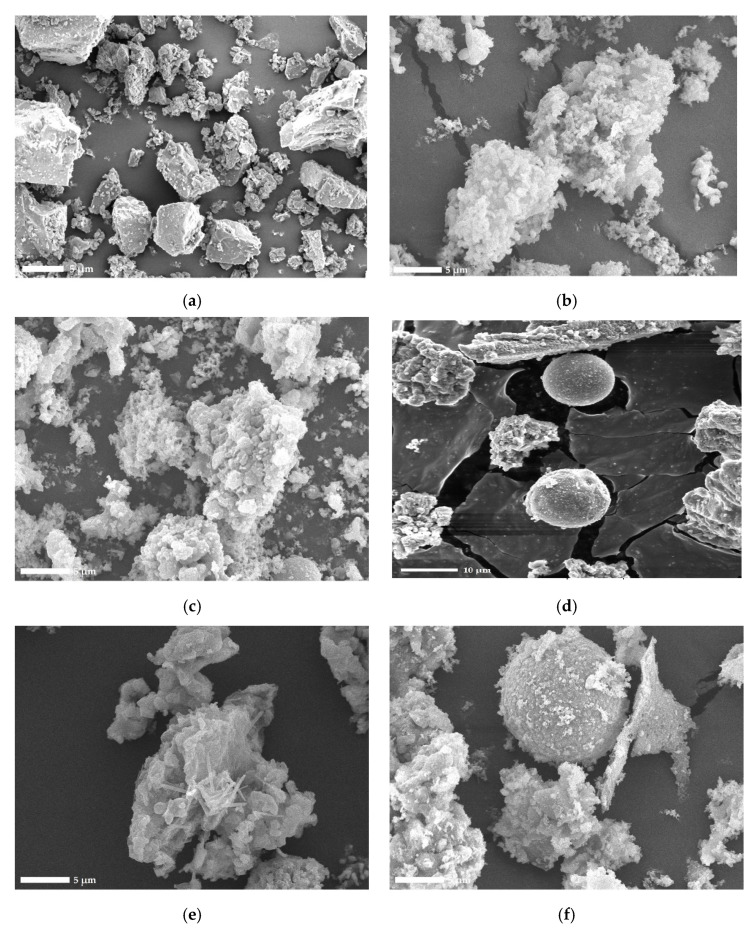
Micrographs of (**a**) CEM sample, (**b**) WBA1 sample, (**c**) WBA2 sample, (**d**) WBA3 sample [17], (**e**) WBA4 sample, (**f**) WBA5 sample (magnification SEM_MAG = 3500×).

WBAs are mainly composed of angular particles of different shapes with a non-uniform structure, which makes them morphologically different from coal fly ash, as they do not contain spherical particles [66]. The difference in morphology between cement and WBA was made clear by the results of the SEM analysis, which are shown as 3500× magnified micrographs (Figure 3). It is visible that the WBA particles were mostly irregular and porous, which is consistent with the claims of authors [45,67,68]. Such particles are more susceptible to water absorption, which may negatively affect the workability of cement composites, as described in Section 3.2. A small number of spherical particles were found in most of the samples. The micrographs also show that these particles were surrounded by minuscule, fluffy structures that increased the specific surface area. The adhesion of nanoparticles to larger angular and spherical particles is characteristic for WBA [69]. It is assumed that during mixing, some water becomes “trapped” in the pores between these particles, leading to an increased water demand when WBA is used as SCM [70]. The irregular particle shape was also confirmed in other studies on the morphology of WBA [39,69,71]. Exceptions were found in the micrographs of WBA3 and WBA5 (Figure 3d,f), in which a small portion of spherical particles with irregular morphology can be seen.

**Figure 4:**
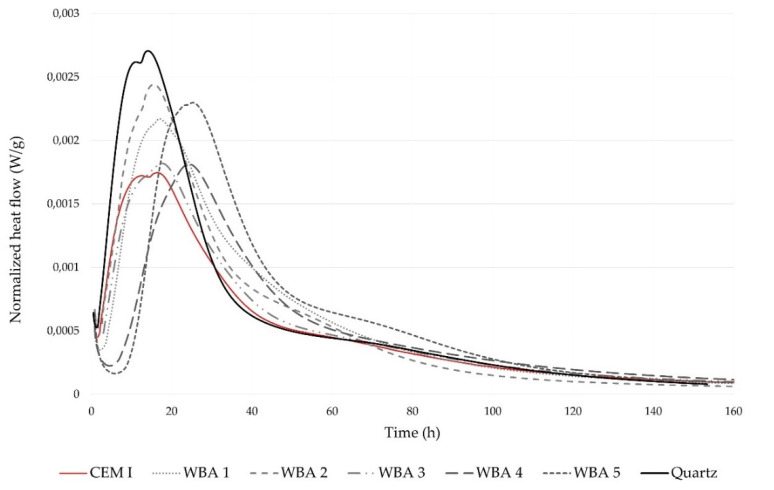
Heat flow of the reference paste (CEM I) without substitution, pastes with 15 wt% WBA, and paste with 15 wt% quartz, obtained by isothermal calorimetry.

**Figure 5:**
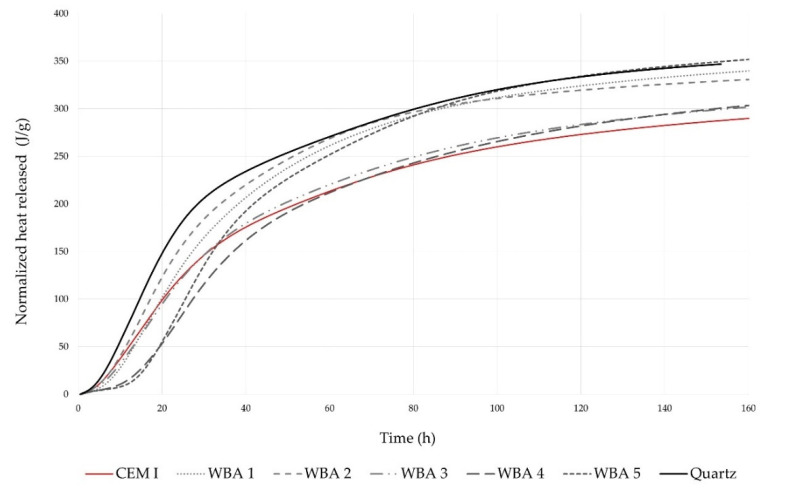
Cumulative heat curves of the reference paste (CEM I) without substitution, pastes with 15 wt% WBA, and paste with 15 wt% quartz, obtained by isothermal calorimetry.

The effect of WBA on binder hydration was studied by monitoring the heat release with isothermal calorimetry. Figure 4 shows the heat release rate of cement paste with 15 wt% WBA, where the flow rate was normalized to g of cement in order to indicate its reactivity. It can be seen that the use of WBA in the cement pastes changed the hydration kinetics: the induction period was prolonged by the addition of WBA, regardless of the type and chemical properties of WBA. This is consistent with previous studies [51,72]. The delay in the induction period of pastes containing WBA, displayed in Figure 4, is explained in the literature by the supersaturated state with calcium oxides [44]. They observed that the presence of the blended coal—wood ashes retard cement hydration, delaying the induction period as the time to reach the supersaturated state was prolonged. The delay in C_3_S hydration is also attributed to the incorporation of alumina and organic ions [73]. Moreover, the addition of WBA resulted in increased peaks, especially in paste blends, in which WBA2 and WBA5 were used. It is known that the addition of pozzolans increases the effective ratio of water to Portland cement and provides an additional surface for nucleation and growth of hydrates, leading to an increase in the heat of hydration [74,75]. Compared to the other samples, WBA5 had a significant amount of pozzolanic oxides, which may have contributed to the increase in heat of hydration. WBA2 had the smallest particles of all the WBAs tested, with half of the particles smaller than 6.10 µm, showing the heat flow curve most similar to the inert quartz. The cumulative heat of hydration after 167 h is shown in Figure 5. The results show higher cumulative heat for the pastes with WBA compared to the reference mix (292.32 J/g cement) as follows (from the highest to the lowest value): WBA5 (354.23 J/g cement) WBA1 (342.06 J/g cement) WBA2 (332.43 J/g cement) WBA4 (306.53 J/g cement) WBA3 (304.49 J/g cement). Based on the hydration analysis, prolonged setting time and lower early compressive strength of composites with WBA can be expected.

### 3.2. Influence of WBA on Fresh Concrete Properties

The results of testing fresh concrete mixes in which 15 wt% of the cement was replaced by WBA and the reference mix CM0 are shown in Table 7. When density was tested, no significant effect of WBA addition on the density of fresh concrete was observed. Although the mixes with WBA showed comparable results to the reference values, the results presented are slightly lower, which was expected because of the lower density of WBAs. Accordingly, the air content increased in parallel with the decrease in density of the concrete mixes, except for mix CM2, which showed the lowest air content. In addition to the density and air content tests, the temperature of the concrete mixes was also observed. The measured temperatures of the fresh concrete were not particularly comparable, which can be attributed to the mixing in the concrete plant at different times of the day and variations in outdoor temperature. The outdoor temperatures during mixing were as follows: 12.7 °C for CM0, 21.2 °C for CM1, 19.6 °C for CM2, 19.2 °C for CM3, 21.9 °C for CM4, and 9.1 °C for CM5. 

The results obtained show that the addition of WBA can lead to a change in the consistency of the concrete mixes. When the mixture CM5 was compared with the reference mix CM0, they showed a similar consistency. Here, the concrete mixes with WBA1 and WBA4 showed a loss of workability. The loss of workability was particularly highlighted in mix CM2, where there was difficulty in molding. Previous characterizations of fly WBAs revealed irregularly shaped and highly porous particles with higher specific surface area and high carbon and free CaO content, all of which can be related to the increased water demand [26,56,66]. Table 5 shows the high LOI values (19.7% for WBA2 and 24.1% for WBA4), which probably caused lower workability of these concrete mixes [76,77]. At the same time, the mixture designated as CM3 showed a slight improvement in consistency.

### 3.3. Influence of WBA on Hardened Concrete Properties

#### 3.3.1. Compressive Strength

**Figure 6:**
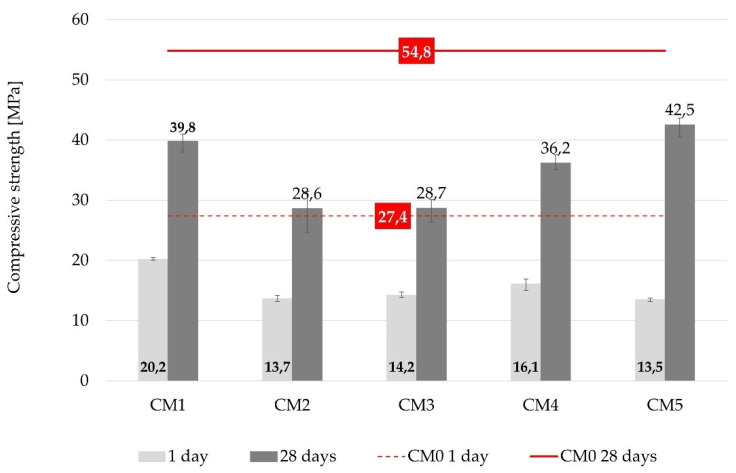
Compressive strength of concrete mixtures after 1 and 28 days.

**Figure 7:**
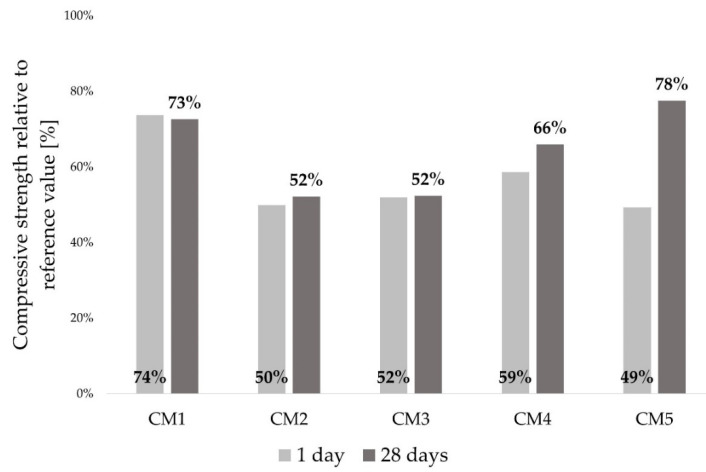
Compressive strength of concrete mixtures relative to the reference values after 1 and 28 days.

The effects of each WBA on mechanical performance of concrete were analyzed based on the demonstrated compressive strength. Thus, the differences between each WBA were reflected in the values of compressive strength of concrete with WBA, evaluated in relation to the control mix CM0 (Figure 6 and Figure 7). From the absolute results of the compressive strength (Figure 6), the reduction in compressive strength was visible, displaying a similar trend for all concrete mixtures after 1 and 28 days. The drop of compressive strength after 1 day differed between 26% (CM1) to 51% (CM5), relative to the control mix. These lower values of early compressive strength can be related to the heat of hydration, where a similar trend was observed (Figure 4 and Figure 5). A stagnant increase in strength was investigated by [17] analyzing six different WBAs and different ash contents at early ages. It was found that the use of WBA reduced the early compressive strength due to high content of free CaO, free MgO, LOI and alkali. At 28 days, the compressive strengths of all the mixtures with WBA content of 15 wt% were lower than that of the reference mix, with downturns ranging from 22% (CM5) to 48% (CM2 and CM3). WBA2 had higher alkali (10.39 wt%) and LOI (19.7 wt%) content compared to the other WBA samples, which can be associated with the lower values of compressive strength [78,79], while WBA3 had coarser particles compared to cement and the other WBAs, which may have led to a decrease in the compressive strength of the CM3 mixture. The negative impact of the reduced value of d_50_ in WBA2 may also be related to compressive strength drop. The compressive strength increased as the concrete aged, but none of the specimens tested achieved a higher compressive strength than the control mix. Other authors have also confirmed that the use of WBA as a cement replacement in concrete can decrease compressive strength values after 28 days [53,67,68,80,81,82,83,84,85,86,87,88].

When analyzing the relative results of compressive strength of concrete with WBA compared to the control mix, an unfavorable effect on strength development was observed for these binary concrete mixes with WBA used as cement replacement at the level of 15 wt%, Figure 7. The effect of WBA was observed in the disruption of cement hydration and change in microstructure, which is related to increased water absorption and permeability [73]. While no prevailing property of WBA was identified to trigger the reduction in compressive strength, the predominant parameter controlling the reactivity and strength of the mixtures was the chemical composition, mainly the sum of pozzolanic oxides, which was inversely related to the hydraulic coefficient. It is noted that the CM2 mixture made with WBA2, which had the lowest content of pozzolanic oxides (21.19%), also had the lowest values for compressive strength. At the same time, the CM5 mix with the topmost pozzolanic activity (58.81%) was the one to reach the highest 28 days compressive strength relative to the control, while showing a larger disparity between the values at 1 and 28 days. A slightly slower increase in strength can be seen for all the mixes containing WBA, which can be attributed to the slightly slower pozzolanic reaction. However, if the reactivity of WBA were to be appraised solely on the basis of hydraulic and pozzolanic properties, the compressive strength of concrete composites containing WBA at a later age would certainly be underestimated [49].

#### 3.3.2. Capillary Absorption

**Figure 8:**
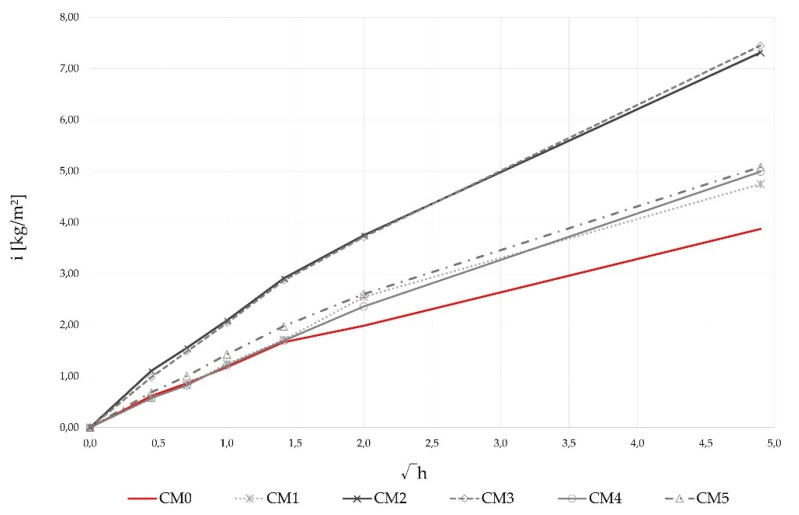
Average water uptake per unit area (i) against square root of the time immersion of concrete samples.

Capillary absorption of precast concrete is not quantified in the EN 1340 standard [89], but specimens with WBA have been tested, as this is one of the main mechanisms of penetration into the concrete surface by which concrete degradation occurs. The capillary absorption of the concrete specimens as a function of root time after 28 days and the values of the sorption coefficients of the concrete mixtures are shown in Table 8 and Figure 8. The increase in the sorption coefficient values compared to the control mix was 56%, on average, for the samples with WBA, from 27% for the CM1 mixture (0.96 kg/m^2^√h) to 96% for the CM3 mixture (1.48 kg/m^2^√h), Figure 8. Based on the given results, the adverse effect of WBA on capillary absorption of concrete samples was observed. As noted by [17,90], moderate cement replacement in the content of 5 wt% to 10 wt% leads to a decrease in the sorption coefficient, while the replacement of 15 wt% cement in this study had an opposite effect. When measuring the increase in weight due to capillary absorption over time, all samples with WBA showed increased values compared to the control mix CM0 (Figure 8). Comparing the mixtures with WBA among themselves, it can be seen that mixtures CM1, CM4, and CM5 showed better results, i.e., lower capillary absorption, than mixtures CM2 and CM3. Moreover, the slope of the CM0 curve decreased after 1.5 √h, which was probably due to the filling of the capillary pores. The same point was not so clear-cut for the samples with WBA, which was probably due to the structure of the cement matrix, i.e., the incorporation of WBA led to an increase in the number of capillary pores [48]. A low Na_2_Oeq content is common in SCMs, but a higher content, as found in the WBA2 specimens, may have a negative effect on the durability of concrete [17].

#### 3.3.3. Total Water Absorption

**Figure 9:**
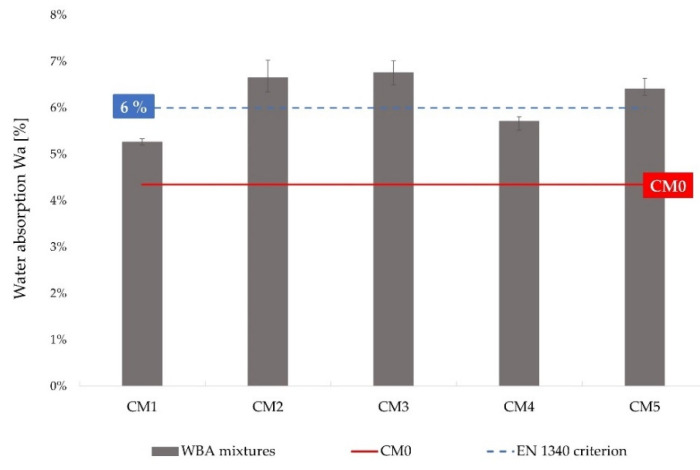
Total water absorption of concrete specimen with WBA in relation to control mix.

The results of testing the water absorption of concrete specimens with WBA are shown in Table 9. The water absorption is expressed as a percentage of the specimen’s own weight, which was determined as the difference between the mass of the water-saturated specimen and that of the dry specimen. It can be seen that the water absorption as a mean of all samples from the mixtures CM2, CM3, and CM5 exceeded the value prescribed by the standard EN 1340 [89], according to which no finished precast concrete element may have a water absorption over 6% by mass, with a maximum ascent of 0.8% (CM3), Figure 9. The feeble results regarding water absorption of CM2 and CM3 mixtures are in agreement with the capillary absorption outcomes, suggesting that the properties of the WBAs used (WBA 2, WBA3, and WBA5) negatively affect the structure of the cement matrix. The control mix CM0 had an average water absorption of 4.3%, which is lower than the prescribed standard value. Although the incorporation of WBAs may have a negative effect on the water absorption of cement composites as the results exceed the maximum allowable value, it is also evident that the predominant factor is the WBA type.

#### 3.3.4. Depth of Penetration of Water under Pressure

**Figure 10:**
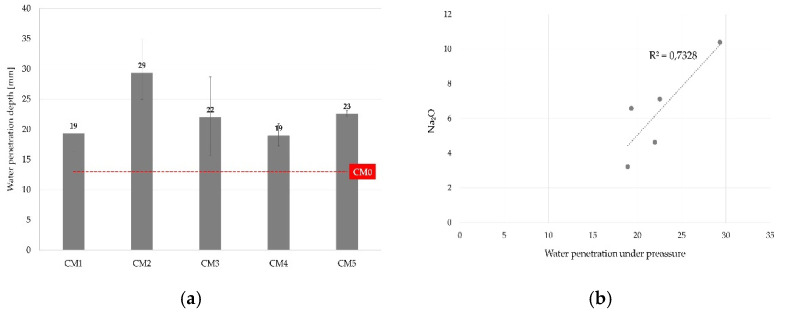
(**a**) Water penetration under pressure; (**b**) correlation between water penetration and alkali content in WBA samples.

The penetration of water under pressure was studied according to EN 12390-8:2019 [91] to assess the water permeability of concrete and to establish a range of values for the maximum depth, depending on the type of WBA and the environment to which the concrete was exposed. The lowest values of water penetration depth of 13 mm were observed in the control mix CM0, followed by mixtures CM1 (19 mm) and CM4 (19 mm). Compared to the control mix, all the mixtures with WBA exhibited lower resistance to water penetration under pressure, where the CM2 mixture displayed the most inferior results (29 mm) (Figure 10a). The alkali content was found to be significant in correlating the WBA properties with the resistance of concrete to water penetration. The coefficient of determination (R^2^) is shown in Figure 10b. In the standard for the application of coal fly ash in concrete EN 450-1:2013 [55], the limit value for alkali content is 5.0 mass percent, and in this case, WBAs with alkali content up to 7.0% had similar resistance to water penetration (mixtures CM1, CM3, CM4). Higher permeability was observed in mixture CM2 prepared with WBA2, in which alkali content was found to be higher than 10.0% by mass. WBA2 also contained a higher proportion of LOI accompanied with diminished workability and a lower content of pozzolanic oxides.

#### 3.3.5. Freeze–Thaw Resistance with De-Icing Salts

When fly ash originating from coal-fired power plants is used in concrete, the decrease in air content is mainly affected by the high LOI and alkali content, the limit of which given by the standard EN 450-1 is often exceeded in WBA concrete, as shown in Table 5 [41,89]. Based on the above, the freeze and thaw resistance of concrete was not expected to improve when WBA was used as a cement replacement. This has been confirmed by previous studies, which found that the use of WBA as a partial cement replacement does not significantly affect the freeze–thaw resistance of concrete [41,92,93]. According to [26,38], the use of WBA in concrete does not negatively affect its freeze–thaw resistance, but concrete mixes containing WBA require a higher air entraining admixture content to achieve the desired amount of pores [26,41,89].

**Figure 11:**
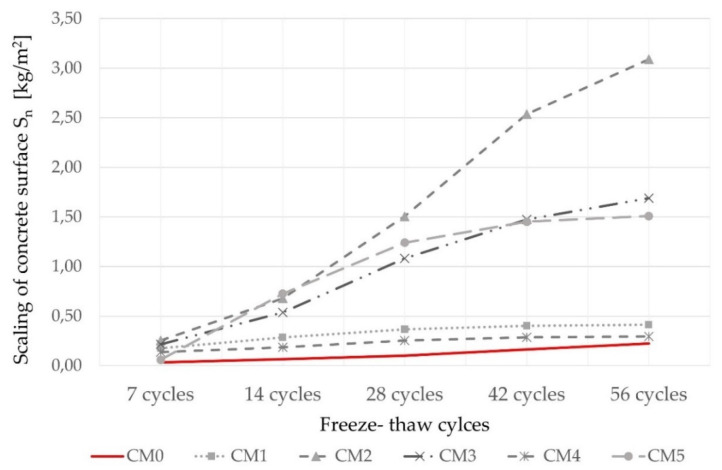
Scaling of test surface due to freeze–thaw attack up to 56 cycles.

In this study, the freeze–thaw resistance of concrete was investigated namely through scaling, i.e., surface weathering, in accordance with CEN/TS 12390-9 [94]. The material loss at the test surface of specimens with 15 wt% WBA due to freeze–thaw attack in the presence of de-icing salts is shown in Table 10 and Figure 11. According to the standard CEN/TS 12390-9, the average scaling after 56 cycles must not exceed the value of 0.5 kg/m^2^ for the precast concrete elements, such as curbs or pavers, to fall within the exposure class XF4 for freeze–thaw risk [50]. In addition, the criterion given by the standard EN 1340 [89] limits the mass loss to an average value of 1.0 kg/m^2^ after 28 freeze–thaw cycles with de-icing salts. From the results demonstrated in Table 10 and Figure 11, only the CM1 and CM4 mixtures met both criteria. Samples from mixture CM2, which had a higher LOI content (19.7%) and the highest alkali content (10.39%), accompanied by the lowest air content (4.4%), showed decreasing freeze–thaw resistance with a maximum value of scaled material of 1.51 kg/m^2^ after 28 cycles and 3.09 kg/m^2^ after 56 cycles. This may be taken as an indicator that a greater amount of air entraining agent was required in this case, confirming the correlation between air instability and freeze–thaw resistance [26].

The specimen with WBA4, which had the highest LOI (24.10%) but also the highest air entrainment (6.5%), showed the least surface deterioration with a minimal mass of scaled material after 28 cycles (0.25 kg/m^2^) and after 56 cycles (0.30 kg/m^2^). For the CM3 mixture, it was not possible to determine which property of WBA had such a significant effect on the scaling of concrete.

### 3.4. Assessing Feasibility of WBA Implementation as SCM in a Large-Scale Industrial Environment

Given the requirements of existing regulations in the concrete industry in Europe, i.e., the lack of standards for the use of WBA in construction products, one of the simplest and most promising ways of using WBA in construction products is covered by System 4 for assessment and verification of constancy of performance. According to Regulation (EU) No. 305/2011 [95], the assessment and verification of the essential characteristics of construction products under System 4 is entirely carried out by the manufacturer, who must set up a factory production control system and self-declare the performance requirements for an authenticating product. With regard to placing concrete products containing WBA on the market, the reliability of the declaration of performance and, thus, the verification of each type of product containing WBA must be ensured. By superimposing the performance requirements given in compliance with the EN 1340 standard and the requirements imposed by the concrete manufacturer, the assessment of concrete mixtures with 15 wt% WBA was carried out to detect a fitting application for each WBA (Table 11).

More extensive requirements are called for by the manufacturer in order to assess the feasibility of using WBA as a secondary raw material in a large-scale industrial batching plant. After implementing a performance-based design along with a detailed characterization of the available WBAs and their compatibility with cement, the need for a trade-off between the amount of WBA and the properties of the finished products became apparent. In order to identify a suitable product for a particular WBA, the mixture design of existing products can be adapted depending on the relevant features and the production process itself. 

Based on the results of freeze–thaw resistance and total water absorption testing, only mixtures CM1 and CM4 met the weathering resistance requirements of EN 1340 (∆m ≤ 1.0 kg/m^2^ after 28 cycles of exposure to freeze–thaw attack in the presence of de-icing salts and w_a_≤ 6% for total water absorption). Higher values of water penetration under pressure were observed in all mixes with WBA, indicating a change in the microstructure of the concrete due to the addition of WBA. Nevertheless, the water penetration in the CM1 and CM4 mixtures was comparable to the control values. Although WBA4 had the highest LOI share (24.1%) and the lowest alkali content (3.22%), the CM4 mixture demonstrated better performance in its hardened state compared to the other mixtures. The most severe deterioration in concrete properties was observed in the CM2 mixture, which contained WBA2 with high LOI and alkali content. Therefore, this type of WBA should be subjected to pretreatment to improve its properties, such as sieving and/or washing, which would lower the alkali content and remove unburnt particles. The WBA3 sample had coarser particles compared to the cement and the other WBAs, which could have a negative effect on the durability properties. Similar findings were also obtained by [17], where the authors concluded that additional mechanical processing, in the form of grinding, is recommended for coarser WBAs before they are used as SCM in cementitious composites.

It is important to point out that the WBA feasibility assessment presented in Table 11 does not include all performance requirements prescribed in EN 1340, i.e., based on this research, investigation of bending strength and abrasion resistance is planned and will be included in future studies. Furthermore, the influence on WBA on the properties of concrete in a hardened state has been highlighted, although maintaining the required workability is an important aspect of industrial production. The workability issue will be addressed by optimizing the overall effect of chemical admixtures to compensate for the increased water requirement, since water-reducing admixtures may be affected by the presence of SCMs such as WBA. The high water absorption due to porous constituents in WBA could be eliminated by selective removal or grinding of these particles. In this case, the industrial sieves and/or mills would be integrated into the concrete production plant or the power plant itself.

## 4. Conclusions

In this study, the behavior of fly WBAs was characterized to implement a sustainable raw material in precast concrete elements as a partial cement substitute and at the same time resolve the problem of industrial waste disposal. The potential of using WBA in cement composites was assessed according to the EN 1340 standard and the limitations obliged by the concrete manufacturer. Based on the data related to compressive strength, capillary uptake, water absorption, water permeability, and freeze–thaw resistance of concrete with WBA, several conclusions can be drawn, which can be used as references for future experimental work based on the investigations carried out:

- After chemical analysis of the WBAs, a predominance of CaO was found at lower density values. Higher LOI and Na_2_Oeq values were the main features of the fluctuating chemical compositions of the WBAs and were primarily reflected in the concrete properties in fresh and hardened states. All the WBA samples exhibited coarser particle size distribution compared to the cement sample, except for the WBA2 sample;
- SEM microscopic images showed a non-uniform structure, inhomogeneous particle surface, and particles of different shapes, with porous, non-spherical particles predominating. The WBA with the most spherical particles was WBA3;
- Based on the results of hydration analysis, prolonged setting time and lower early compressive strength of composites with WBA are expected due to the prolonged induction time, regardless of the type and chemical properties of WBA;
- Partial cement replacement with WBA did not significantly affect, i.e., reduce, the density of fresh concrete. The inconsistency in the number of pores in fresh concrete is presumably caused by higher LOI share in WBAs, affecting the stability of air content;
- As a result of the consistency tests, all concrete mixes showed the same trend of increased water demand. Utilization of WBA as a partial substitute for cement unfavorably alters the slump of concrete. The reduced workability caused by the replacement of cement by WBA is strongly dependent on the WBA type;
- Using WBA as a partial cement replacement up to 15 wt% reduced the compressive strength of concrete. An indicator of achieving higher compressive strengths with partial cement replacement is a higher sum of pozzolanic oxides;
- By adding WBA to concrete as cement replacement, capillary absorption, water absorption, and the penetration depth of water under pressure increased. In addition to the influence of alkalis, WBA could lead to a change in microstructure and thus to certain properties;

Compared to the reference mixture, all mixtures with WBA exhibited diminished resistance to freeze–thaw attack in the presence of de-icing salts. Nevertheless, the mixtures with WBA1 and WBA4 showed satisfactory freeze–thaw resistance after 56 cycles, thus meeting the more stringent requirements of the manufacturer.

WBA can be used as a pared down replacement of cement in concrete mixes while maintaining concrete quality, although all the properties studied have demonstrated high dependence on the type of WBA used. In addition to prior chemical analysis of WBA and assessment of particle size distribution, it is advisable to determine whether a particular WBA is suitable as a partial replacement for cement or would it be more applicable as a sand substitute. While the application of WBAs generally degrades concrete properties, some WBAs were proved to enhance the freeze–thaw resistance, i.e., to give satisfactory results in terms of water permeability and water absorption. When using WBA in the industrial production of concrete elements, end-use and finished product requirements should be considered and attention should be paid to the design of the concrete composition (amount of superplasticizer, air-entraining admixture, etc.). With respect to the examined mechanical and durability properties of concrete with WBA in the industrial environment, additional pre-treatment of some WBA would be needed in order to ensure a constant quality of the raw material. 

Even with satisfactory results, these figures still provide a fragmentary understanding of the new potential material in the construction industry with no recommendations for practice. Therefore, strict and regular quality control is necessary at all stages of production.

## Figures and Tables

**Table 1:** Properties of fly WBAs and the origin of power plants.

WBAs			Combustion Properties		Biomass
WBA ID	Visual check	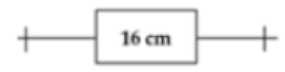	Technology	Temperature	Wood species
WBA1	Fine, grayish, powdery material visually resembling cement, no impurities found.	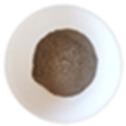	Biograte	680 °C	mixed wood
WBA2	Fine powdery material of light gray color visually resembling cement, no impurities were found.	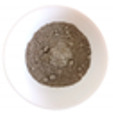	grate pulverized fuel combustion	700–750 °C	beech, oak, hornbeam, mixed wood
WBA3	Fine powdery material of taupe color visually resembling cement. No impurities were found.	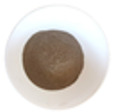	grate combustion	550 °C	beech, oak, fir, and spruce
WBA4	Fine powdery material of dark gray color with a smaller percentage of impurities.	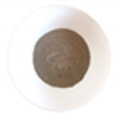	grate combustion	980 °C	beech, oak, hornbeam, poplar, ash tree
WBA5	Fine powdery material of dark gray color, small amount of charred wood and larger particles were found.	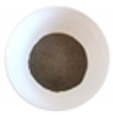	grate combustion	700 °C	beech, poplar, pine, mixed wood

**Table 2:** Concrete mix design.

Mix ID			CM0	CM1	CM2	CM3	CM4	CM5
Binder	Cement [kg]		420	357				
	WBA	[kg]	-	63				
		[wt%]		15				
Aggregate	0–4 mm [%]		47					
	4–8 mm [%]		26					
	8–16 mm [%]		27					
Admixtures	Superplasticizer [%]		0.50	w/b = 0.44				
	Air entrainer [%]		0.10					

**Table 3:** Test methods for WBA assessment.

Property	Test Period	Unit	Standard
Density	Prior to mixing	g/cm^3^	ASTM C-188-17
Chemical composition		wt.%	ISO/TS 16996:2015
Loss on ignition (LOI)			ASTM D 7348-13
pH value		-	EN ISO 10523:2005
Heat of hydration		J	HRN EN 196-11:2019
Particle size distribution		μm	Laser diffraction particle size analysis
Particle morphology		-	Scanning electron microscope (SEM)

**Table 4:** Test methods for assessment of concrete in fresh and hardened states.

State	Property	Test Period	Unit	Standard
Fresh concrete	Density	Immediately upon mixing	kg/m^3^	EN 12350-6: 2019
	Temperature		°C	EN 12350-1: 2019
	Air content		%	EN 12350-7: 2019
	Consistence of fresh concrete by the slump test		mm	EN 12350-2: 2019
Hardened concrete	Compressive strength	After 1 and 28 days of curing	MPa	EN 12390-3: 2019
	Capillary absorption	After min. 28 days of age	kg/m^2^h^0.5^	EN 13057: 2003
	Total water absorption		%	EN 1340:2003
	Depth of penetration of water under pressure		mm	EN 12390-8: 2019
	Freeze–thaw resistance with de-icing salts	After 7,14, 28, 42, and 56 cycles	kg/m^2^	CEN/TS 12390-9:2016

**Table 5:** Chemical composition of WBAs and cement.

WBA Samples		WBA1	WBA2	WBA3	WBA4	WBA5	Mean Value	EN 450-1 [55]	CEM I 42.5 R
pH value	-	12.90	13.51	13.04	12.89	12.85	13.04	-	12.66
LOI (950 °C)	wt.%	6.2	19.7	8.80	24.1	5.1	12.78	9.0	5.5
P_2_O_5_		4.64	3.01	2.50	1.46	2.30	2.78	5.0	0.02
Na_2_O		0.84	1.30	0.56	0.92	3.01	1.33	-	0.63
K_2_O		8.73	13.82	4.78	3.50	6.24	7.41	-	1.16
CaO		42.75	51.68	58.24	54.99	18.97	45.33	-	51.72
MgO		5.56	4.57	3.81	2.86	3.91	4.14	4.0	1.49
Al_2_O_3_		4.84	2.45	3.96	5.11	10.30	5.33	-	6.33
TiO_2_		0.23	0.11	0.17	0.29	0.90	0.34	-	0.18
Fe_2_O_3_		2.62	1.71	1.9	2.83	3.61	2.53	-	3.28
SiO_2_		22.15	17.03	21.78	26.76	44.90	26.52	-	30.79
MnO		0.73	0.50	0,47	0.25	0.63	0.53	-	0.02
SO_3_		6.93	3.42	1.84	1.06	5.24	3.70	3.0	4.33
Na_2_Oeq		6.58	10.39	3.71	3.22	7.12	6.20	5.0	1.39
SiO_2_ + Fe_2_O_3_ + Al_2_O_3_		29.61	21.19	27.64	34.70	58.81	34.39	70	40.4
(CaO + MgO)/SiO_2_		2.18	3.30	2.85	2.16	0.51	2.20	-	1.73

**Table 6:** Physical properties of WBAs and cement.

Sample ID	Density [g/cm^3^]	d_50_ [µm]
WBA1	2.67	92.95
WBA2	2.61	6.10
WBA3	2.59	129.52
WBA4	2.47	61.58
WBA5	2.21	84.48
CEM	3.01	24.18

**Table 7:** Properties of concrete mixtures in fresh state.

	Mix ID	CM0	CM1	CM2	CM3	CM4	CM5
Properties							
Density [kg/m^3^]		2340	2260	2250	2230	2200	2230
Temperature [°C]		16.2	25.2	23.1	22.2	25.5	14.9
Air content [%]		5.0	5.2	4.4	6.5	6.5	4.9
Consistence—Slump [mm]		220	165	70	250	160	210

**Table 8:** Sorption coefficient of specimens with WBA and reference mix.

Mix ID	CM0	CM1	CM2	CM3	CM4	CM5
S (kg/m^2^√h)	0.76	0.96	1.44	1.48	1.01	1.01
Min. value	0.67	0.89	1.37	1.43	0.93	0.87
Max. value	0.83	1.07	1.52	1.52	1.06	1.12

**Table 9:** Total water absorption of samples with WBA and control mix.

Mix ID		CM0	CM1	CM2	CM3	CM4	CM5
Total water absorption W_a_ [%]	Specimen 1	4.4	5.2	6.4	6.9	5.9	6.4
	Specimen 2	4.3	5.6	6.9	6.9	5.6	6.6
	Specimen 3	4.3	5.0	6.7	6.6	5.6	6.3
	Mean value	4.3	5.3	6.7	6.8	5.7	6.4

**Table 10:** The material loss at the test surface of specimens with 15 wt% WBA due to freeze–thaw attack in the presence of de-icing salts.

Mix ID	CM0	CM1	CM2	CM3	CM4	CM5
LOI [%]	5.5	6.2	19.7	8.80	24.1	5.1
Air content [%]	5.0	5.2	4.4	6.5	6.5	4.9
Na_2_O_eq_ [%]	1.39	6.58	10.39	3.71	3.22	7.12
Scaling after 28 cycles [kg/m^2^]	0.10	0.37	1.51	1.08	0.25	1.24
Scaling after 56 cycles [kg/m^2^]	0.22	0.41	3.09	1.69	0.30	1.51

**Table 11:** Assessment recap of concrete mixes with WBA in relation to performance requirements.

		Relevant Property	Standard Requirement		CM1	CM2	CM3	CM4	CM5
Concrete manufacturer		Consistency—Slump [mm]	100–150		165	70	250	160	210
		Minimum air content [%]	4		5.2	4.4	6.5	6.5	4.9
		Compressive strength [MPa]	C 35/45		39.8	28.6	28.7	36.2	42.5
		Water penetration under pressure [mm]	≤15		19	29	22	19	23
		Freeze–thaw resistance with de-icing salts [kg/m^2^]	∆m ≤ 0.5 (56 cycles)		0.41	3.09	1.69	0.3	1.51
	EN 1340		∆m ≤ 1.0 (28 cycles)		0.37	1.51	1.08	0.25	1.24
		Total water absorption [%]	≤6		5.3	6.7	6.8	5.7	6.4
		Abrasion resistance [cm^3^/cm^2^]	≤21		Long-term research scope				
		Bending strength [MPa]	Characteristic value	Minimum value					
			≥3.5	2.8					
			≥5.0	4.0					
			≥6.0	4.8					

## Data Availability

Not applicable.
